# The Impact of L Arginine’s Anti-Apoptotic, Anti-Inflammatory, and Anti-Fibrotic Effects in Modulating Diabetic Cardiomyopathy: The Role of microRNA-155b in the Diagnosis of Diabetic Cardiomyopathy

**DOI:** 10.3390/ijms27104546

**Published:** 2026-05-19

**Authors:** Shimaa E. Soliman, Heba M. Iraqy, Omnia I. Ismail, Mohamed E. Ali, Tohamy Anwar Tohamy, Ayman Sabry Yassin Al Sayed, Maha Ali, Mai A. H. Abouelenin, Zahraa Azab Mohamed Abouelatta, Tarek A. Salem, Eman Radwan, Hayam G. Sayyed, Nessren M. Abdel-Rady

**Affiliations:** 1 Department of Pathology, College of Medicine, Qassim University, Qassim 51452, Saudi Arabia; 2 Medical Physiology Department, Faculty of Medicine, Assiut University, Assiut 71515, Egyptnessren@aun.edu.eg (N.M.A.-R.); 3 Department of Human Anatomy and Embryology, Faculty of Medicine, Assiut University, Assiut 71515, Egypt; 4 Department of Microbiology and Immunology, Faculty of Pharmacy, Al-Azhar University, Assiut 71524, Egypt; 5 Medical Research Center, Assiut University, Assiut 71516, Egypt; 6 Department of Microbiology and Immunology, Faculty of Medicine, Al-Azhar University, Assiut 71524, Egypt; 7 Department of Medical Biochemistry and Molecular Biology, Faculty of Medicine, Assiut University, Assiut 71515, Egypt; 8 Medical Biochemistry and Molecular Biology Department, Faculty of Medicine, Menoufia University, Menoufia 32511, Egypt; 9 Department of Clinical Pathology, Faculty of Medicine, Menoufia University, Menoufia 32511, Egypt; 10 Department of Biochemistry, Sphinx University, New Assiut 71684, Egypt; 11 Basic Medical Science Department-Medical Physiology, Badr University in Assiut, Assiut 51711, Egypt; 12 Medical Physiology Department, Sphinx University, New Assiut 71684, Egypt

**Keywords:** myocardium, L-arginine, diabetic cardiomyopathy, cardiac hypertrophy, left ventricular ejection fraction (LVEF), left ventricular fractional shortening (LVFS), biomarkers, toll-like receptor-4 (TLR4), pro-inflammatory interleukin 1 beta (IL-1β), interleukin 6 (IL-6), anti-inflammatory interleukins (IL-4; IL-13), apoptotic markers (bcl-2; bax) and real-time PCR

## Abstract

Diabetic cardiomyopathy (DC) is a serious health issue. MicroRNA-155b expression dysregulation might be involved in the fibrotic cycle in DC. L-Arginine (l-arg) is reported to have a preferable impact on the cardiovascular system. We aimed to understand the pathogenesis of DC and to detect the potential protective effect of l-arg through modulation of apoptosis, inflammation, fibrosis, and miR-155b expression. This study comprised four groups of forty adult male rats (10 rats in each group): diabetics, l-arg diabetics, l-arg, and controls. Blood glucose, systolic blood pressure (SBP), diastolic blood pressure (DBP), heart rate (HR), body weight, and cardiac hypertrophy index (HW/BW ratio) were assessed. Echocardiographic assessment of left ventricular ejection fraction (LVEF) and left ventricular fractional shortening (LVFS) was done. Expressions of toll-like receptor-4 (TLR4), pro-inflammatory interleukin 1 beta (IL-1β), interleukin 6 (IL-6), anti-inflammatory interleukins (IL-4, IL-13), apoptotic markers (bcl-2, bax) and microRNA-155b were measured by real-time PCR. Myocardial light, electron microscopic and morphometric studies were performed. Results showed a significant decrease in cardiac hypertrophy (HW/BW = 0.0030 ± 0.0002 mg/g), echocardiographic parameters (LVEF = 54.12 ± 1.628% and LVFS = 20.40 ± 0.541%), hemodynamic parameters (HR = 411.0 ± 9.684 bpm, SBP/DBP = 84 ± 4.998/60 ± 3.062 mmHg) and downregulation of the expression of IL-4, IL-13, IL- 1β, IL-6 and TLR4 in the l-arg diabetic group compared to diabetic rats. Additionally, restoration of normal appearance of most cardiac myofibrils, intact blood vessels, decreased cardiac fibrosis and upregulation of bax expression were observed. Expression of microRNA-155b increased by 0.007 for each gram increase in blood glucose (>1.45, it showed 100% specificity and 96.7% sensitivity). In conclusion, microRNA-155b upregulation is associated with enhancement of the transcription of inflammatory cytokines and apoptotic genes. L-arginine may be a useful protective strategy for DC through modulation of apoptosis, inflammation, and fibrosis, in addition to regulating the expression of miR-155b.

## 1. Introduction

Over 463 million individuals worldwide suffer from diabetes mellitus (DM), one of the most common chronic metabolic diseases [1]. It grows exponentially, especially among obese individuals. DM is strongly associated with interdisciplinary problems like neuropathy, nephropathy, and retinopathy. Almost all the related diabetic microvascular and macrovascular pathophysiological complications are indeed a predictable outcome. The interactions of various diabetes-induced disorders, such as inflammation, apoptosis, hyperglycemia, and insulin resistance, are directly blamed for these complications [2].

Among the most affected systems by DM is the cardiovascular system. Diabetic cardiomyopathy (DC) is characterized by myocardial structural distortion, diastolic dysfunction, and systolic dysfunction, which ultimately leads to heart failure [3].

Unfortunately, DC pathogenesis has not yet been scrutinized. Apoptosis and inflammation are greatly implicated. A key factor in the development of diabetes mellitus and its progression to diabetic cardiotoxicity is programmed cell death [4,5]. Overexpression of the toll-like receptor-4 (TLR4) occurs in diabetes [6] and enhances inflammatory cytokine manufacturing; in particular, interleukin 1 beta (IL-1β) and interleukin 6 (IL-6) [7]. Their effects are thought to be hindered by interleukin 13 (IL-13) and interleukin 4 (IL4), which have anti-inflammatory, metabolic, and myocardial remodeling consequences [8].

microRNA-155b is encoded by the MIR155 gene and plays a potentially harmful role in cardiovascular disease progression [9]. Its expression dysregulation was noticed during the fibrotic cycle of cardiomyopathy in diabetic models [10,11]. However, the association between this disruption and the various pathological arbitrators participating in DC is still questionable.

The amino acid L-arginine (L-arg) has favorable effects on the cardiovascular system because nitric oxide, a potent vasodilator, is derived from it [12]. Plasma arginine concentrations in diabetic patients are low, which eventually leads to diabetic vascular dysfunction [13]. However, not much is known about how L-arg affects DC.

Thus, using echocardiographic, histological, immunohistochemical, and electron microscopic analyses, the current research was created to examine the potential protective impact of L-arg on the hearts of albino rats with diabetes caused by STZ. In addition, we aim to establish that microRNA-155b is dysregulated in DC and associated with the modulation of inflammatory, apoptotic, and fibrotic events contributing to the pathogenesis of DC.

## 2. Results

### 2.1. Fasting Blood Glucose Levels and Final Body Weights (FBWs)

Rats given a high-fat diet in both the diabetic and l-arg + STZ-D groups showed significantly higher mean FBW compared to the chow-diet-fed control rats. Moreover, the diabetic group had a higher FBW relative to the l-arg group; however, there was no discernible difference between the two groups who received l-arg treatment.

The two groups given STZ (diabetic and l-arg + STZ-D, being higher in the diabetic group) had noticeably greater fasting blood glucose levels than the other two groups (Table 1).

### 2.2. Cardiac and Hemodynamic Parameters

#### 2.2.1. Cardiac Hypertrophy Index (HW/BW Ratio), SBP, DBP and HR (Table 1)

The diabetic and l-arg + STZ-D groups showed a significantly higher HW/BW value compared to controls. Moreover, the HW/BW ratio of diabetic rats was noticeably higher than that of the l-arg group. However, compared to the diabetic rats, the l-arg + STZ-D group had a much lower HW/BW ratio.

When compared with the control rats, the l-arg and l-arg + STZ-D groups had considerably lower SBP values. Additionally, compared to the l-arg and diabetic groups, the mean SBP was considerably lower in the l-arg + STZ-D group. On the other hand, the SBP of the diabetic group was much greater than that of the l-arg group.

Compared to the control and l-arg groups, the diabetic group’s mean DBP was noticeably greater. Conversely, compared to the other research groups, the l-arg + STZ-D group showed the lowest DBP value. However, there was no discernible difference in DBP between the control and the l-arg groups.

Both the diabetic group and the l-arg + STZ-D group had considerably greater heart rates (being higher in diabetic rats) than the control group; meanwhile, the l-arg group had significantly lower values.

#### 2.2.2. Echocardiographic Parameters; LVEF and LVFS (Table 1, Figure 1):

LVEF and LVFS were considerably lower in the diabetic and l-arg + STZ-D groups than the other two groups. More remarkably, the l-arg + STZ-D group showed significantly higher LVEF and LVFS values than the diabetic group.

#### 2.2.3. Myocardial Fold Expression of Apoptotic Genes (bax and bcl-2), Different Cytokines (IL1β, IL-6, IL-4 and IL-13) and TLR4 mRNAs

The diabetic group showed a substantially higher value of bax and a lower value of bcl-2 than the control and l-arg groups. Furthermore, compared to the control and l-arg groups, there was a considerable upregulation of bax in the l-arg + STZ-D group; however, this expression was much lower than that of the diabetic group (Table 2).

Remarkably, both the l-arg and l-arg + STZ-D groups demonstrated a large increase in bcl-2 when compared with the control rats, and the l-arg + STZ-D group also showed a significant rise in bcl-2 when compared with the diabetic rats. Nevertheless, there was no discernible difference between the l-arg and l-arg + STZ-D groups. Furthermore, fasting blood glucose correlated positively with the expression of bax and negatively with bcl-2 expression (Table 3).

The diabetic group exhibited a marked increase in both IL-1β and IL-6 myocardial expression compared to the control and l-arg groups. Similarly, the l-arg + STZ-D group showed significant upregulation of both cytokines’ expression when compared with the l-arg group and considerable upregulation of IL-6 expression when compared with the control rats. However, compared to the diabetic group, the expression of both cytokines was much reduced in the l-arg + STZ-D group (Table 2).

The expression of IL-4 and IL-13 was remarkably high in the diabetic and l-arg + STZ-D groups compared to the other two groups. Meanwhile, the expression of both cytokines was markedly reduced in the l-arg + STZ-D group when compared with the diabetic rats (Table 2).

Comparing the diabetic and l-arg + STZ-D groups to the other two groups, TLR4 expression was also markedly elevated; nevertheless, it was significantly downregulated in l-arg + STZ-D compared to diabetic rats. Furthermore, a strong positive correlation existed between the expression of TLR4 and blood glucose, and all measured interleukins (Table 3).

#### 2.2.4. Myocardial Expression of microRNA-155b

**Figure 2:**
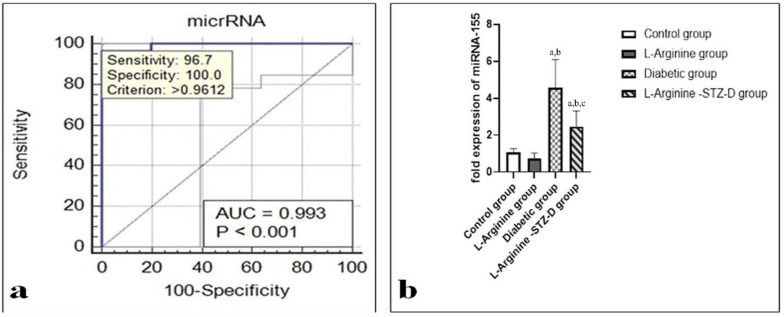
(**a**) Receiver operating characteristic (ROC) curve for analysis of fold change expression of microRNA-155b; AUC = area under the curve. (**b**) Fold expression of microRNA-155b in different studied groups. Data are represented as mean ± standard deviation. STZ-D, streptozotocin-induced diabetic. a: significant compared with controls. b: significant compared with the L-Arginine group. c: significant compared with the Diabetic rats. Significant means *p* < 0.05.

Receiver operating characteristic (ROC) curve for microRNA-155b, at a cutoff value of 1.45, showed 100% specificity and 96.7% sensitivity with AUC = 0.993 (Figure 2a).

Both the diabetic and l-arg + STZ-D groups exhibited significantly higher expression of microRNA-155b (being lower in the l-arg + STZ-D group) when compared with the other two groups (Table 2, Figure 2b).

MicroRNA-155b expression was markedly positively correlated with TLR4, the measured pro-inflammatory interleukins, anti-inflammatory interleukins and pro-apoptotic bax; nevertheless, it lacks correlation with bcl-2 (Table 3).

**Figure 3:**
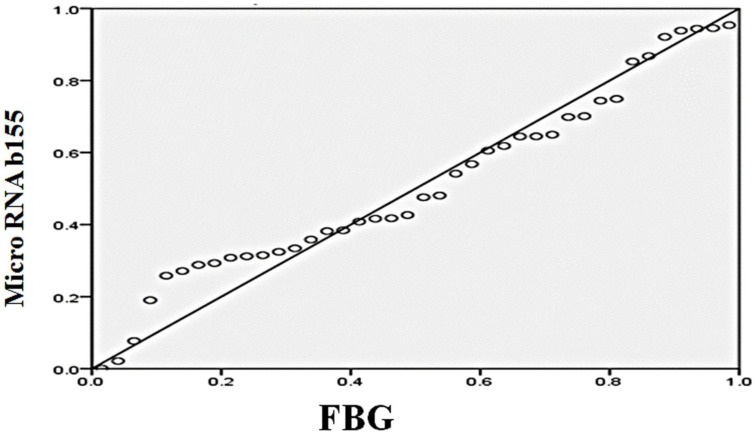
Regression analysis between myocardial expression of microRNA-155b and fasting blood glucose (FBG).

The simple linear regression analysis used to predict the myocardial expression of microRNA-155b based on fasting blood glucose levels showed a significant regression equation (F = (1.38) = 81.973, *p* < 0.000) with an R2 of 0.683. There was a significant positive relationship between fasting blood glucose and the expression of microRNA-155b (beta coefficient; 0.827, *p*: 0.000) as the myocardial expression of microRNA-155b increased by 0.007 for each gram increase in blood glucose (Figure 3).

#### 2.2.5. Histological Results

**Figure 4:**
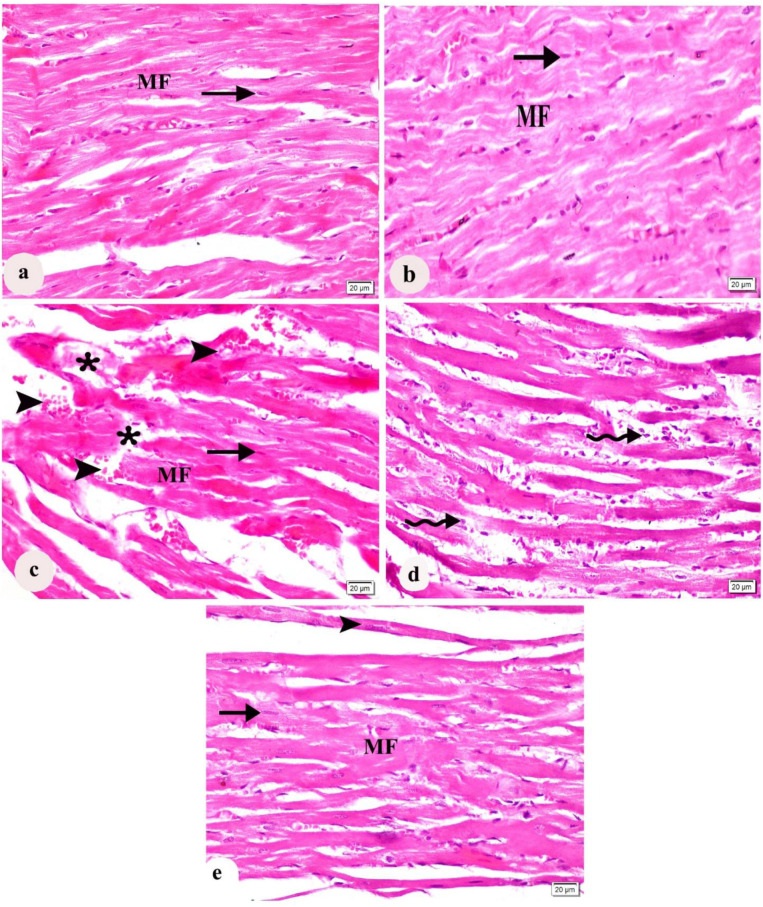
A longitudinal section of the heart tissue photographed under a microscope. The cardiac myofibers (MF) were in a regular arrangement with oval centrally located nuclei of cardiomyocytes (arrow) in control and L-Arginine groups (**a,b**). (**c**) The diabetic group showed irregularly arranged MF with oval central nuclei of cardiomyocytes (arrow), interruption of normal arrangement of myofibers (star) and extravasation of RBCs (arrow head). (**d**) The diabetic group showed cellular infiltration (curved arrow). (**e**) The L-Arginine + STZ-D group showing regularly arranged MF with oval nuclei of myocardial cells (arrow) and MF showed thinning (arrow head) (H&E × 400, 20 µm is the scale bar).

In both the control and l-arg groups, microscopic examination of haematoxylin and eosin-stained longitudinal slices of rat hearts showed the characteristic architecture of regular organization of the cardiac myofibres and oval centrally positioned nuclei of cardiomyocytes, as shown in (Figure 4a, Figure 4b, respectively). Conversely, the diabetic group had degeneration in some regions of the cardiac myofibers as well as disruption of their usual arrangement. Also, hemorrhage and extravasation of RBCs were observed (Figure 4c). Additionally, the diabetic group showed signs of inflammatory cellular infiltration (Figure 4d). In the l-arg + STZ-D group, the majority of cardiac myofibers showed a restoration to their typical histological appearance. However, apparent thinning and atrophy of some cardiac myofibers were found (Figure 4e).

**Figure 5:**
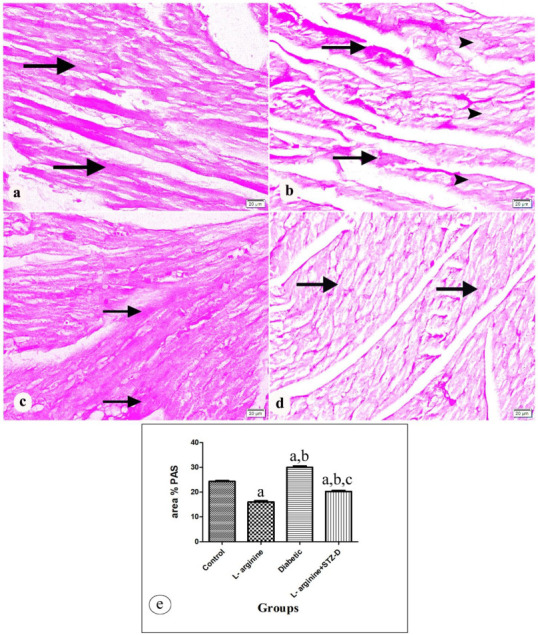
A longitudinal segment of the heart tissue photographed under a microscope in (**a**) control rats displaying normal distribution of glycogen and positive PAS response (arrow), and (**b**) the L- Arginine group, revealing positive PAS response (arrow) within some areas. Other areas show decreased PAS reaction (arrow head). (**c**) The diabetic group showing increased PAS staining (arrow). (**d**) The L-Arginine + STZ-D group showing apparent decreased PAS reaction (arrow) compared to control and diabetic groups. (**e**) Bars represent mean ± standard error (SE) of area percent (%) of PAS staining among the studied groups [a: significant compared with controls. b: significant compared with the L-Arginine group. c: significant compared with the Diabetic rats. Significant means *p* < 0.05] (PAS × 400, scale bar = 20 µm).

PAS-stained longitudinal cardiac slices examined under a light microscope revealed that the control rats’ myocardium had a normal distribution of glycogen (Figure 5a). The l-arg groups displayed positive PAS reaction in some areas. However, other areas showed decreased PAS reaction (Figure 5b). In contrast, increased PAS stain in diabetic rats when compared to the controls (Figure 5c). The l-arg + STZ-D group revealed decreased positive PAS reaction relative to control and diabetic rats (Figure 5d). A statistically significant variation in the mean area percent of PAS values (*p*-value < 0.0001) between the studied groups was detected via one-way ANOVA analysis (Figure 5e, Table 4).

**Figure 6:**
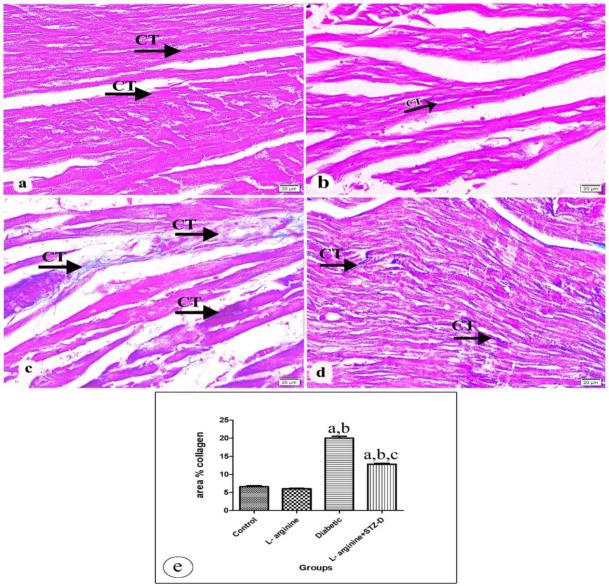
A photomicrographic picture of heart tissue in (**a**) the control rats revealing normal few interstitial collagen fibers between cardiac myofibers (arrow), and (**b**) the L-Arginine group showing similar pattern found in control group. (**c**) The diabetic group showing increased amount of collagen deposition compared with control group (arrow). (**d**) The L-Arginine + STZ-D group showing interstitial collagen fibers (arrow) less than that in the diabetic group but more than appear in the control group. (**e**) Bars represent mean ± standard error of area percentage of the deposited collagen among studied groups [a: significant compared with controls. b: significant compared with the L-Arginine group. c: significant compared with the Diabetic rats. Significant means *p* < 0.05] (Masson’s trichrome × 400, scale bar = 20 µm).

The cardiac slices were stained with Masson’s trichrome to show how diabetes and concurrent l-arg treatment affected collagen deposition. Both control and l-arg rats’ heart slices had a normal distribution of interstitial collagen fibers between cardiac myofibers (Figure 6a and Figure 6b, respectively). However, in the STZ-induced diabetic group (Figure 6c) showed an increased amount of collagen deposition compared to controls. Heart sections of the l-arg + STZ-D group revealed fewer interstitial collagen fibers than those in the diabetic rats, but more than those in the controls (Figure 6d). Statistically significant difference (*p*-value < 0.0001) in the average area percentages of collagen deposition between studied groups was detected via One–way ANOVA analysis (Figure 6e, Table 4).

#### 2.2.6. Morphometric Results (Cardiomyocyte Cross-Sectional Area)

**Figure 7:**
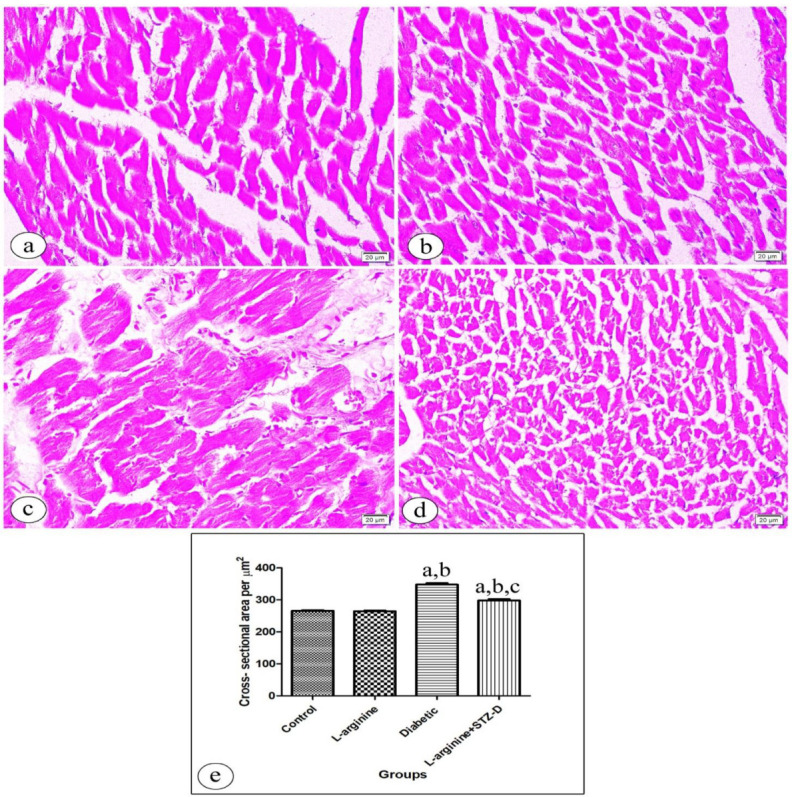
A transverse section of the heart tissue captured in a photomicrograph, revealing apparent increased cross-sectional area of cardiomyocyte in the control, L-Arginine and diabetic groups (**a**, **b** and **c** respectively). (**d**) The L-Arginine + STZ-D group showing apparent decreased cardiomyocyte cross-sectional area compared to diabetic group. (**e**) The mean ± standard error of cross-sectional area per μm^2^ among the studied groups is represented by bars [a: significant compared with controls. b: significant compared with the L-Arginine group. c: significant compared with the Diabetic rats. Significant means *p* < 0.05] (H&E × 400, scale bar = 20 µm).

The mean cross-sectional areas of cardiomyocytes of all groups were assessed in H&E-stained transverse sections (Figure 7a–d) and utilized as an indicator of cell size. The diabetic group (347.7 ± 19.6 µm^2^) revealed a considerable increase within the cardiomyocytes cross-sectional area compared to the control group (265.17 ± 10.36 µm^2^), with *p*-value < 0.0001. However, the l-arg + STZ-D group (297.7 ± 21.9 µm^2^) revealed a considerably lower mean cross-sectional area when compared to the diabetic group (347.7 ± 19.6 µm^2^) with *p*-value < 0.001. The l-arg group’s mean cross-sectional area (264.9 ± 8.1 µm^2^) did not differ significantly from that of the control group (Figure 7e).

#### 2.2.7. Results of Electron Microscopy

**Figure 8:**
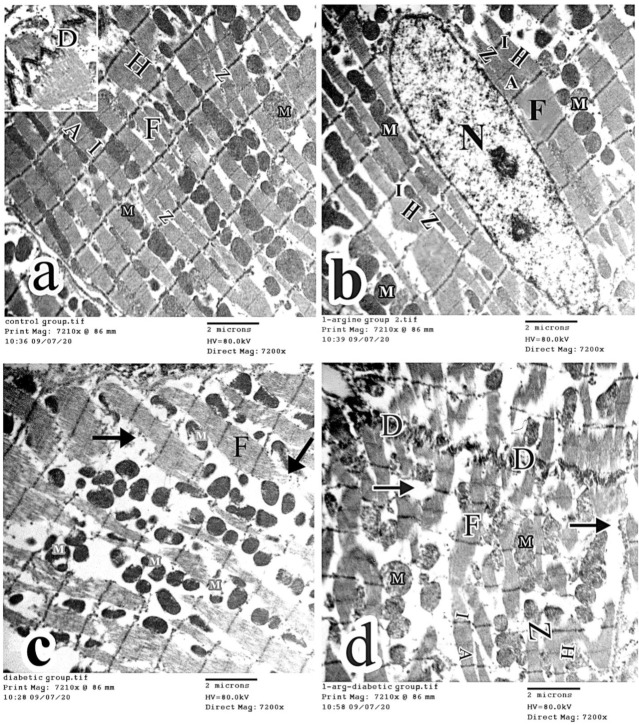
The cardiomyocyte electron micrographs in (**a**) control rats displaying long parallel arrays of myofibers (F) that consist of sarcomeres with alternating dark (A) bands, light (I) bands, a well-demarcated Z line (Z), and an H line (H). The intercalated disk (D) and many normal mitochondria (M) arranged in rows between myofibers are seen. (**b**) The L-Arginine group showing normal histological structure of the myofibers with oval euchromatic nucleus (N). (**c**) The diabetic group showing degenerated parts of the myofibers (arrow) and destroyed mitochondria (M). (**d**) The L-Arginine + STZ-D group showing partial restoration of normal structure of myofibers (F), intercalated disk (D) and mitochondria (M). Areas of myofibers loss (arrow) are observed (transmission electron microscope (TEM) × 7200, scale bar = 2 µm).

**Figure 9:**
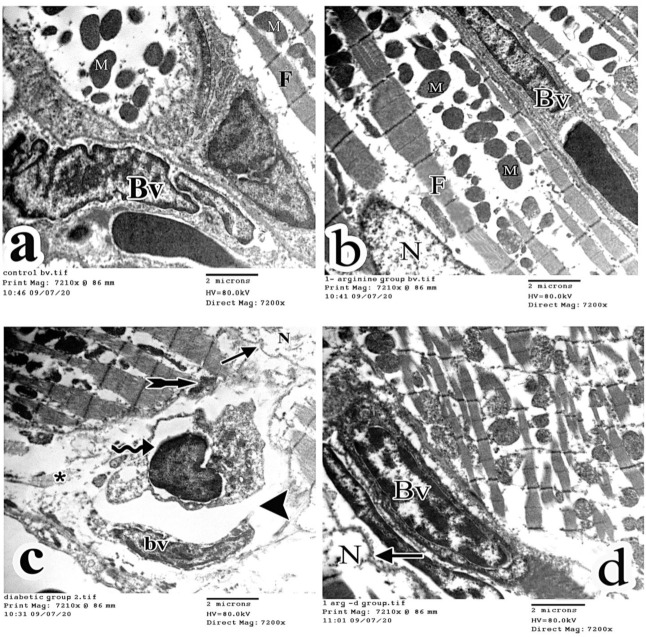
Blood vessel electron micrographs displaying blood vessels’ normal endothelium in control rats and L-Arginine group (**a** and **b** respectively). (**c**) Micrographs in diabetic rats showed discontinuous blood vessels’ endothelium (arrow head). An irregular outline (arrow) of cardiomyocyte nucleus (N), disfigured intercalated disc (tailed arrow), macrophage infiltration (curved arrow) and collagen fibers deposition (asterisk) were observed. (**d**) The L-Arginine + STZ-D group showing blood vessels’ normal endothelium and indentation (arrow) of the membrane of cardiomyocyte’s nucleus (N) (BV: blood vessels, TEM × 7200, 2 µm is the scale bar).

Myocardial ultrastructural analysis in control and l-arg groups showed a normal arrangement of the cardiac myofibers that consisted of sarcomeres with alternating dark and light bands. A well-demarcated Z line appeared in the middle of light bands. An H line was observed in the middle of dark bands. Rows of mitochondria in the perinuclear area within the myofibrils and a well-demarcated intercalated disk were noticed (Figure 8a and Figure 8b, respectively). On the other hand, the examination of the diabetic group demonstrated the degeneration of some myofibers with interruption of their arrangement and destruction of some mitochondria. The irregular outline of the cardiomyocyte nucleus and a disfigured intercalated disk were also observed. Moreover, macrophage infiltration and collagen fiber deposition were seen (Figure 8c). In the l-arg + STZ-D group, partial restoration of the myofibers to their original structure, intercalated disk and mitochondria were noticed. However, areas of loss of myofibers were present and indentation of the nuclear membrane of cardiomyocyte was observed (Figure 8d). Regarding the ultrastructure of the blood vessels, the intact blood vessels’ regular endothelium was found in control and l-arg groups (Figure 9a and Figure 9b, respectively). However, the diabetic rats revealed discontinuous endothelial lining of the blood vessels (Figure 9c). Interestingly, the l-arg + STZ-D group showed normal intact blood vessels (Figure 9d).

## 3. Discussion

Diabetic cardiomyopathy is a serious health issue characterized by morphological and functional cardiac muscle inefficiency that ultimately leads to heart failure [14]. According to the American heart association in 2020, eighty percent of diabetic patients globally died from DC [15]. The great problem is that its precise pathogenesis is not fully clarified. Thus, it is very necessary to find new model medications or diagnostic biomarkers in DC.

In our study, by performing echocardiographic measurements, we found that DM had positively triggered cardiac impairment and hypertrophy as shown by the increased HE/BW ratio and decreased LVEF and LVFS. However, all cardiac injuries were attenuated by the administration of l-arginine.

We hypothesized that this improvement in cardiac function observed in l-arg-treated groups can be explained by l-arginine’s ability to regenerate the inadequate cardiac nitric oxide (NO). Nitric oxide is synthesized from l-arg and plays an important role in the regulation of angiogenesis, vascular remodeling, and has a protective effect on ischemia–reperfusion injury [16]. Additionally, NO is known to inhibit apoptosis by decreasing caspase-3 and enhance endothelial cell proliferation and migration [17].

Administration of l-arginine reinforces the anti-inflammatory condition, enhances glucose metabolism and insulin sensitivity [18,19]. In addition, other studies found neuronal nitric oxide synthase (nNOS) and inducible nitric oxide synthase (iNOS) immunoreactivity were lower in the diabetic group when compared with the controls [20,21,22]. The potential explanation was that DM damages the heart’s tissue histologically leading to a decrease in nNOS and iNOS expression [23]. In DM, a reduction in the bioavailability of NO increased fatty acid production, and a reduction in ATP generation triggered endothelial dysfunction, leading to the elevation of reactive oxygen species and oxidative stress in the cells [24]. Furthermore, DC impairs lipid peroxidation and disturbs oxidant-antioxidant balance leading to decreased cardiomyocytes NO [25]. On the other hand, Nagareddy et al. and Atta et al. revealed that the iNOS gene expression fold was markedly elevated in diabetic rats due to oxidative damage to the heart muscles [26,27].

In this study, diabetic rats showed a marked elevation in the measured heart rate when compared with control and l-arg diabetic treated groups. This is in accordance with the previous study of Yu and Zheng who stated that DC is associated with cardiac arrhythmia [28]. This could be explained by the fact that DC is associated with inflammatory, apoptotic, and oxidative stress imbalance conditions. The significant decrease in HR in diabetic rats after treatment with l-arg in the present study could also be explained through Mary’s baroreceptor reflex [29]. There was a significant decrease in both SBP and DBP in the l-arg STZ group when compared to the diabetic group, and these results could be partly explained by the potent l-arginine vasodilation, which is mediated by the induction of endothelial nitric oxide synthesis [30] that raises 3′,5′-cyclic-guanosine monophosphate and cGMP-dependent protein kinase activity, which mediates vascular smooth muscle cell relaxation [31]. Moreover, the antioxidant effect of NO counters the endothelial dysfunction created by the accumulating free radicals with DM.

Also, the present work revealed that the cross-sectional area of cardiac myofibers was enlarged in the diabetic group when compared with the control rats. By contrast, concomitant l-arg administration appeared to prevent cardiac hypertrophy in diabetic rats.

This was in line with the studies of Fang et al. and Cheng et al., who noticed that left ventricular hypertrophy is commonly detected in DC patients [32,33]. A possible explanation for the current observations is that left ventricular hypertrophy is a cardiac way to compensate against various pathophysiological mechanisms and discrepancies in hormonal signaling, which is associated with diabetic stress [34]. The heart goes through extensive cardiac remodeling which leads directly to the abnormal proliferation of cardiomyocytes [35]. This response was originally intended to reduce this stress effect and maintain contractile performance; however, if persistent, it ends up causing hypertrophy and ultimately causing cardiac failure [36]. Additionally, DM affects the cardiomyocytes’ proteins such as titin, which assists in myocardial passive tension and stiffness and ultimately leads to hypertrophy [24]. Additionally, cardiac hypertrophy has been associated with elevated TNF-α and cardiac NF-κB expression, which reduce cardiomyocyte degradation and boost protein synthesis [37]. Additionally, the fatty acid oxidation rate was increased, leading to lipid buildup and, ultimately, hypertrophy of the heart [38]. This eventually causes myocardial rigidity and hypertrophy by lessening myocardial soluble guanylate cyclase and cyclic GMP myocardial content, thus closing down the protein kinase G’s shielding property [39].

The increased number of interstitial collagen fibers in the diabetic group detected by Masson’s Trichrome, while being reduced in the l-arg + STZ-D group in the present study, is supported by Felício et al., who reported interstitial fibrosis and capillary membrane thickening in DM. Many studies have tried to explain the pathophysiological mechanisms of DM-induced cardiac fibrosis [40,41]. In addition, ultrastructural examination of the myocardium in the diabetic group revealed collagen fiber deposition; this can be explained by the fact that hyperglycemia encourages cardiomyocyte myofibroblast formation [42], and epicardial epithelial cells transition to myofibroblasts [43]. This is in line with our results, as giving l-arginine to rats after induction of DM attenuated the DC hypertrophy and fibrosis effects. Tan et al. and Rizk et al. proved that l-arg could lower blood glucose, modulate insulin tolerance and reestablish the profibrotic and anti-fibrotic factors stability and thus counter the occurrence of myocardial hypertrophy [13,18].

Diabetic cardiomyopathy begins with diastolic dysfunction that becomes worsened over time with systolic dysfunction, thereby aggravating the condition and leading to heart failure and arrhythmias due to defective mitochondrial function [40], irregular Ca^2+^ homeostasis [43], and autonomic neuropathy [44,45]. This was demonstrated in the present work as destruction of mitochondria under electron microscopic evaluation.

Extravasation of red blood cells and hemorrhage was noticed in the diabetic group under light microscopic examination and ruptured blood vessels under electron microscopic examination in the current study may be explained by the rise in reactive oxygen species (ROS) and NOS uncoupling, which causes blood vessel endothelial dysfunction. The NO-dependent antioxidative, antithrombotic, and antiadhesive effects become weaker as NO bioavailability diminishes [20].

The increased accumulation of glycogen detected by PAS staining in the diabetic group, which was decreased in the l-arg + STZ-D group, is in line with Kohli et al. [46]. L-arginine increases insulin release and attenuates insulin resistance, which in turn induces net protein synthesis and glucose utilization in skeletal muscles [46,47,48].

For a long time, the study of pathophysiological mechanisms that explain morphological and functional changes in cardiomyocytes associated with DC has been considered a serious issue. According to Li et al. apoptosis or programmed cell death that appears in the heart during cardiac remodeling may be crucial in the transition from compensatory hypertrophy to cardiac failure [49]. To the best of our knowledge, the current work is the first one to identify apoptosis in cardiomyocytes as a primary cause of DC, which is observed to improve following l-arg administration. In the present work, the STZ-induced diabetic group demonstrated positive caspase-3 immunoreaction in cardiomyocyte cytoplasm with a significant increase in bax gene expression and a significant decrease in the expression of bcl-2 gene within the cardiac tissue homogenate. However, this was reversed in l-arg + STZ-administrated rats, where there was a marked increase in the expression of bcl-2 gene and a considerable decrease in bax gene expression within the cardiac tissue homogenate, with negative caspase-3 immunoreaction. This is in accordance with a previous study [50].

According to our findings, fasting blood glucose significantly positively correlated with the expression of the bax gene, while it negatively correlated with bcl-2 gene expression in cardiac tissue homogenate. These findings are attributed to high blood glucose and DM-associated insulin resistance, which acts as physiological trigger-promoting factors that disrupt the transcription of the apoptotic markers—mainly, the bax gene with the anti-apoptotic bcl-2 gene being downregulated, and decreasing NO production and endothelial nitric oxide synthase activity [51,52]. This impressively induces the activation of mitochondrial cytochrome C dependent caspase 3, which ultimately leads to cardiomyocyte programmed cell death [51]. In addition, a significant decrease in cardiac free radical content, cellular defense against damage caused by ROS, and antioxidant defense enzyme activity have been linked to necrotic cell death in STZ-induced diabetic rats [36,53,54]. Our results showed that l-arginine has an anti-apoptotic effect that is in accordance with the previous studies in various cell types, for example, hepatic cells [55] and skin fibroblasts [56]. This is the first in vivo study so far highlighting the cardio-protective role of l-arg against cardiomyocytes programmed cell death by altering bax and bcl-2 mRNA in heart tissue from diabetic rats.

In the present study, the myocardial expression of cytokines that mediate inflammation such as IL-1β and IL-6 mRNA was measured, and they were both significantly (*p* value < 0.01) upregulated in the diabetic group when compared with the controls. Further evidence for these conclusions came from the detection of inflammatory cellular infiltration in H&E-stained sections under a light microscope. So, myocardial inflammation aids in providing another possible pathophysiological mechanism to understand the etiology of DC. This goes in line with previous studies that stated that there was high expression of both myocardial and circulating interleukin-1β [57] and interleukin-6 [58] in diabetic rat models.

The persistent high blood glucose and β oxidation associated with diabetic cardiomyopathy increase free radical formation, which activates the inflammasome that initiates cardiac cell injury and chemotaxis of T lymphocytes, especially Th1, which produce IL-1 and IL-6 [51] and finally trigger cellular apoptosis and heart failure [59]. The overall diabetic rise in pro-inflammatory cytokines can stimulate fibroblasts in cardiomyocytes and initiate a vicious cycle between metabolic and immunological dysfunction, which ends in fibrosis and hypertrophy [60].

It is interesting to note that this work was the first to identify l-arg’s possible therapeutic function against DC-related cardiomyocyte inflammatory disorders. When l-arg was given to diabetic rats in the (l-arg + STZ-D) group, the production of pro-inflammatory cytokines IL-1β and IL-6 in the heart tissue homogenate was considerably downregulated (*p* value < 0.01) in comparison to the diabetic rats. This is consistent with the findings of earlier research that suggested the suppressive effect of l-arg on IL-1β in lipopolysacriade-induced inflammatory diarrhea [12] and IL-6 in pregnant mice [61].

Surprisingly, in our results, instead of the anti-inflammatory effect of IL-4 by suppression of pro-inflammatory cytokines production [62], there was a significant upregulation in the cardiac tissue homogenate fold change expression of the anti-inflammatory cytokine IL-4 in diabetic rats when compared with the control and l-arg + STZ-D groups. These findings could be explained by the synergistic effect of both DM and cardiac fibrosis associated with DC, which was established in the diabetic group. DM stimulates the Th2 cells to naturally produce IL-4 to reestablish normal insulin sensitivity by improving Akt phosphorylation and adjusting hyperglycemia [63]. When interstitial cardiac fibrosis develops in elderly mice, IL-4 is upregulated, indicating cardiac pro-fibrotic actions [64]. Il-4 was also found to encourage the collagen deposition among fibroblast cells and even proliferate the fibroblast cells themselves [65].

Similarly, the diabetic group’s cardiac tissue homogenate expression of IL-13 was considerably higher (*p* value < 0.01) than that of the control and l-arg + STZ-D groups. This goes in line with the study of Amit et al., who found that the left ventricular myocardial expression of the IL-13 gene was enhanced with DM [8]. Serum IL-13 was substantially increased with insulin resistance and hyperglycemia [66]. Also, DM complicated with heart failure exhibited a positive relation between serum IL-13 and the prognosis of diabetic patients [67].

Such rise in IL-13 could be a compensatory mechanism against DC, as IL-13 triggers the signal transducer and activator of transcription (STAT3) and (STAT6) expressed in the surface of the cardiomyocyte, which in turn induces phosphorylation processes of IRS-1 and AKT, and the latter mediates intracellular insulin effects [68]. Additionally, IL-13 causes pancreatic beta cells to secrete more insulin [69]. Furthermore, Wodsedalek et al. stated that IL-13 is a new motivator for cardiomyocytes activity and regeneration after injury [70].

All these explanations successfully support our results of significantly decreased (*p*-value < 0.01) IL-4 and IL-13 myocardial expression in l-arg diabetic-administrated rats when compared to diabetic groups, although they were both still significantly (*p*-value < 0.01) overexpressed when compared with the control rats. These contrasting results might be explained by the potent anti-inflammatory and antioxidant effect of l-arg that can be used as an adjuvant treatment for inflammatory bowel disease [12]. These results also support our research hypothesis that l-arg could decrease both DM and cardiac fibrosis; the main key features of DC; suggesting its therapeutic effect against DC.

Despite the fact that IL-4 and IL-13 are typically categorized as anti-inflammatory cytokines, there is growing evidence that they may potentially have pro-fibrotic effects [71] in heart tissue under long-term metabolic stress situations like diabetes [72]. Therefore, rather than being a fundamentally protective strategy, their increased expression in the diabetic group may be a compensatory response to inflammation and fibrosis. The overall abatement of cardiac inflammation and fibrosis may be reflected in the decrease in IL-4 and IL-13 expression after L-arginine administration [73,74].

In the same way, cardiac tissue homogenate expression of TLR4 was considerably higher (*p* value < 0.01) in the diabetic group when compared with the control and l-arg STZ-D groups. Furthermore, strong positive correlations existed between the TLR4 gene expression and blood glucose and the different studied cytokines (IL-1β, IL-6, IL-4 and IL-13). This goes in line with the study of Degirmenci et al., who found that TLR4 receptor gene overexpression is obvious in myocardial tissue, endothelial cells, macrophages, and smooth muscle cells as a vital anti-inflammatory agent [75]. It mainly induces insulin resistance and increases DM severity and cardiac fibrosis [75,76]. However, cardiac tissue homogenate expression of TLR4 was significantly downregulated (*p*-value < 0.01) in l-arg diabetic-administrated rats when compared with the diabetic rats, although it was still significantly (*p*-value < 0.01) overexpressed when compared to the control group. This is in line with the previous study that stated that l-arg downregulates TLR4 through its anti-inflammatory and antioxidant effects [12].

In this study, we aimed to explore how the cardiomyocyte microRNA-155b expression relates to molecular pathways involved in DC. To our knowledge, this is the first study to examine myocardial microRNA-155b expression in this context. Our findings show that microRNA-155b expression is closely associated with markers of inflammation and apoptosis, processes that are well-known to contribute to cardiac fibrosis and hypertrophy. In particular, microRNA-155b expression was positively correlated with TLR4, the studied cytokines (IL-1β, IL-6, IL-4, and IL-13), and the pro-apoptotic marker bax (all *p* < 0.001).

We also observed a strong positive correlation between blood glucose levels and microRNA-155b expression (β = 0.827, *p* < 0.001), pointing to a possible link between hyperglycemia and its myocardial expression. Consistent with this, microRNA-155b levels were significantly higher in the diabetic group compared with both control and L-arg + STZ groups (*p* < 0.01).

While our study does not establish a direct mechanism, previous work (e.g., Guo et al.) has shown that reduced microRNA-155b expression is associated with protection against cardiac hypertrophy, as well as changes in apoptosis and fibrosis [77] through the TGF-β1/Smad2 pathway [78]. Taken together, our findings support the idea that microRNA-155b may be involved in pathways relevant to the development of DC, although further mechanistic studies are needed.

Furthermore, we evaluated the potential diagnostic performance of microRNA-155b. At a cutoff value of 1.45, it showed high specificity (100%) and sensitivity (96.7%) in cardiac tissue homogenates. Although promising, this observation needs validation in larger and independent studies before firm conclusions can be drawn [79]. Nowadays, myocardial damage and cardiac dysfunction are commonly diagnosed using well-established cardiac biomarkers including cardiac troponins (cTnI and cTnT) and natriuretic peptides (BNP and NT-proBNP) [80]. Nevertheless, rather than the underlying molecular pathways, these indicators mainly indicate myocardial injury or hemodynamic stress. On the other hand, microRNA-155b might shed further light on the pathophysiological mechanisms of diabetic cardiomyopathy, such as fibrosis, inflammation, and apoptosis.

This study does not directly indicate a causal molecular mechanism, despite the fact that the current findings show substantial relationships between L-arginine administration, microRNA-155b expression, inflammatory cytokines, and apoptotic markers. To validate the molecular significance of this microRNA in diabetic cardiomyopathy, more research incorporating microRNA-155b knockdown, inhibition, or overexpression trials is required.

In addition, ROC analysis showed that microRNA-155b had a strong diagnostic performance (AUC = 0.993); however, these results should be regarded cautiously. The risk of model overfitting and overestimating diagnostic accuracy may be increased by the comparatively small sample size and the lack of an independent validation cohort. Therefore, to validate the diagnostic utility of microRNA-155b in diabetic cardiomyopathy, bigger sample sizes and independent validation cohorts are needed in future research. In order to assess its potential clinical utility, future research should directly compare its diagnostic performance with conventional biomarkers.

## 4. Materials and Methods

### 4.1. Drugs and Chemicals

Streptozotocin (STZ) and L-arginine (l-arg) were acquired from Sigma-Aldrich Co., St. Louis, MO, USA. Fermentas (Glen Burnie, MD, USA) provided the microRNA kits.

Real-time PCR kits were obtained from Invitrogen, Carlsbad, CA, USA. The SYBR Green Mix was purchased from Bioline, Memphis, TN, USA. All other chemicals and reagents were of analytical grade and obtained from standard commercial suppliers.

### 4.2. Animals and Diets

The ethics committee at the Faculty of Medicine, Assiut University, Egypt, approved the animal studies used in this work, and they were carried out in accordance with the globally recognized guidelines for the Care and Use of Laboratory Animals (local approval number: 17300531). Forty adult male albino rats weighing 300 ± 20 g were bought from Assiut University’s Faculty of Medicine Animal House, Egypt. They were kept in roomy cages with a 12 h light-dark cycle and a temperature of 22 ± 2 °C. Rats were categorized into four groups at random after becoming acclimated. There were ten rats in each group. Rats in the first two groups were fed ad libitum with commercial pellets, which served as the basal diet, while in the last two groups, the rats received a high-fat diet (HFD) [14]. The Department of Animal Nutrition and Clinical Nutrition, Faculty of Veterinary Medicine, Assiut University in Egypt developed both the basic diet and HFD, which were maintained for ten weeks.

### 4.3. Experimental Design

Rats were randomly split into four groups, each with ten rats: (1) The control rats: 10 rats received no treatment. (2) The L-arginine (l-arg) group: 10 rats administered l-arg (1g/kg) orally by gastric gavage for 4 weeks [15]. (3) The diabetic group (STZ-induced diabetic group): STZ (30 mg/kg diluted in distilled water) was administered intraperitoneally (i.p.) once to ten rats [16]. Using an AC-CU-CHEK glucose meter (Roche Diagnostics, Paris, France) to measure tail vein blood glucose, diabetes was verified 48 h later. The study included animals that had blood glucose levels ≥ 2 g/L (250 mg/dL), and those with blood glucose levels lower than the previous measure were injected with the same dose of STZ for a second time [16]. (4) The L-arginine diabetic (l-arginine + STZ-D) group: 10 STZ-induced diabetic rats received l-arg for 4 weeks in the same previous doses.

Serum glucose levels after an overnight fast were measured once a week throughout the experiment by employing an ACCU-CHEK glucose meter (Roche Diagnostics, Paris, France) to measure blood glucose in the tail vein. An automated balance S/SI-2002 (Fisher Scientific, Carlsbad, CA, USA) was used to assess body weight. An Electrosphygmomanometer Pneumatic Pulse Transducer (Model LE 5001 Pres-sure Meter, Panlab, Holliston, MA, USA) was used to monitor the heart rate and blood pressure in conscious rats before and after the experiment ended, using the indirect tail cuff method. Prior to the recordings, the animals were pre-warmed in a metal chamber at around 35 °C and given 30 min to acclimatize. At the same time of day (8–10 a.m.), three consecutive readings were taken. The mean of the three measurements of systolic blood pressure (SBP) and diastolic blood pressure (DBP) values was determined.

### 4.4. Echocardiographic and Hemodynamic Parameters

After being anesthetized by 1.5% isoflurane, rats’ echocardiography was done by using a MyLab 30CV ultrasound (Biosound Esaote S.p.A., Genoa, Italy), as described previously [17]. LV ejection fraction and LV fractional shortening were determined.

### 4.5. Detection of Cardiac Hypertrophy and Sample Collection

Animals were sacrificed when the experiment was over. Rats’ hearts were taken out and placed in saline then on paper before being measured for heart weight (HW) using a digital scale. The cardiac hypertrophy index (HW/BW ratio) in mg/gm was calculated [18]. After opening the thoracic cavity, dissection of the heart was performed. The connective tissue and adhering adipose tissue were cleansed. Each rat’s heart was split in half longitudinally. One part was used for histopathological examination. The other one was immediately saved in liquid nitrogen and then transferred and stored at −80 °C until use.

### 4.6. Expression of miRNA-155b

#### 4.6.1. miRNA Extraction and Reverse Transcriptase

The miRNA easy Mini Kit (Qiagen, Hilden, Germany; Cat. No. 217004) was used to extract miRNAs from cardiac tissue. The Biotech nanodrop was used to measure the concentration and purity of RNA(BioTech, Winooski, VT, USA). The poly A tail of short non-coding miRNA was increased utilizing the poly A polymerase enzyme (New England Biolabs, lpswich, MA, USA; Cat. No. M0276L) Following reverse transcription, cDNA was synthesized using the Thermo Scientific Revert Aid Reverse Transcription kit (Applied Biosystems, Foster City, CA, USA; Cat. No. 4368814).

#### 4.6.2. Quantitative Real-Time Polymerase Chain Reaction (PCR) Analysis for miRNAs

The procedure was carried out starting with a initial denaturation step at 95 °C for 7 s, then 40 cycles using a 7500 rapid real-time PCR (Applied Biosystems, Carlsbad, CA, USA) consisting of denaturation at 95 °C for twenty seconds, annealing, and extension for 60 s at 59 °C. The fold change 2^−ΔΔCT^ technique was used to calculate the relative transcript levels of microRNA-155b. The primer was obtained from Invitrogen (Carlsbad, CA, USA), and U6-snRNA served as an internal control [19].

### 4.7. Expression of mRNA of the Studied Genes and Real-Time PCR

Following the manufacturer’s instructions, total RNA was isolated from cardiac tissue using a TRIZOL reagent (Invitrogen, Carlsbad, CA, USA), and reverse transcribed into cDNA using a reverse transcription kit ((Applied Biosystems, Foster City, CA, USA; Cat. No. 4368814). SensiFAST SYBR® Lo-ROX Kit (Bioline, Memphis, TN, USA; Cat. No. BIO-94020) and the ABI 7500 Sequence Detection System (v2.0.6) (Applied Biosystems, USA) were used for real-time PCR. A three-step cycling process (denaturation for thirty seconds at 94 °C, annealing for thirty seconds at 60 °C, and extension for one minute at 72 °C) was employed for 35 cycles following an initial denaturation stage lasting five minutes at 94 °C. Table 5 lists the primer sets that were used. Relative gene expression was measured using the 2^−ΔΔCT^ technique and normalized to the level of GAPDH (internal control).

### 4.8. Histological, Electron Microscopic, and Morphometric Study

#### 4.8.1. Examination by Light Microscope

The specimens underwent a dehydration procedure with alcohol series after being fixed in 10% formaldehyde and then embedded in paraffin. Tissues were sectioned (3–5 sections/specimen) using a microtome (MICROM HM 340E, Walldorf, Germany) with 5 μm thin sections. The sections were stained with Hematoxylin and Eosin (H&E) using Masson’s Trichrome (MT) for histopathology to examine the deposition of collagen fibers, and Periodic acid–Schiff (PAS) stain to evaluate glycogen distribution [20].

#### 4.8.2. Electron Microscopic Examination

Specimens were fixed with 2.5% glutaraldhyde + 4% formaldehyde for 2 h after being cut to around 3 mm^3^; then, distilled water was added 3 times for ten minutes for washing the primary fixation. The 1% Osmium Tetroxide was added for about one hour, followed by washing three times for ten minutes. A sequence of dehydration was completed. After being immersed in a pure Epon-Araldite combination, the tissues underwent polymerization. An ultra-microtome was used to cut semi-thin slices (1 micron), which were then stained using 2% aqueous toluidine blue. Using a Leica ultracut UCT (Deerfield, IL, USA), ultra-thin sections (0.1 micron) were then mounted and dyed using lead citrate and uranyl-acetate [21]. The transmission electron microscope (TEM) (“Jeol” E.M.-100 CX11; Tokyo, Japan) at the Electron Microscopic Unit of Assiut University was used to examine the stained ultra-thin sections.

#### 4.8.3. Morphometric Examination

The cross-sectional area of the cardiomyocyte was measured using transverse Hematoxylin and Eosin-stained sections at ×400 magnification. Using computerized image analyzer software (Leica Q 500 MCO, v2.1; Wetzlar, Germany) connected to a camera and to a Leica universal microscope at the Human Anatomy and Embryology Department, Faculty of Medicine, Assiut University, Egypt, a line was manually drawn around the circumference of cardiomyocytes with central nuclei [22,23]. Image J software version 1.48 was used to calculate the percentage of collagen area in Masson’s Trichrome-stained sections and the percentage of PAS area in PAS-stained sections. For every specimen in every group, the average of 10 readings was calculated.

### 4.9. Statistical Analysis

The SPSS software version 16 (SPSS Inc., Chicago, IL, USA) was used to analyze the data. The mean ± SD was used to express the results of quantitative data. The Shapiro–Wilk test was used to determine whether the data distribution was normal before statistical analysis. For multiple group comparisons, Tukey’s post hoc test was employed after one-way ANOVA to determine if there were statistically significant differences between three or more independent groups. Pearson’s correlation was used to detect correlations between the measured variables. Regression analysis was carried out between myocardial expression of microRNA-155b and fasting blood glucose. Receiver operating characteristic (ROC) curve for microRNA-155b was performed. Statistical significance was defined as *p* < 0.05 [24].

## 5. Conclusions

Collectively, our study highlighted the marked role of hyperglycemia, diabetes-induced local inflammation, and apoptosis in the pathogenesis of structural and functional features of DC. It is a progressive cardiac muscle failure, characterized by a number of cardiac morphological and functional abnormalities, including left ventricular diastolic and systolic dysfunction, myocardial fibrosis, cardiomyocyte hypertrophy, and apoptosis. These DC traits are predictive markers of death in diabetic individuals. Additionally, it clarifies the potential benefits of l-arg administration for DC. It offers a potentially helpful management approach for DC, as it improves myocardial fibrosis, myocardial degeneration, cardiomyocyte hypertrophy, and apoptosis. It enhances iNos expression and suppresses cardiac inflammation. It plays an integral role in endothelial homeostasis. Finally, it is the first study to show the role of microRNA-155b in DC, which is believed to enhance the transcription of inflammatory cytokines and apoptotic genes. Those factors induce myocardial structural changes and dysfunction. One important approach for preventing cardiac injury in the diabetic population may be to target these signaling pathways. This study implies that L-arginine may have cardioprotective effects in an experimental model of diabetic cardiomyopathy; nevertheless, before considering its therapeutic use, more experimental and clinical research is necessary.

## Figures and Tables

**Figure 1:**
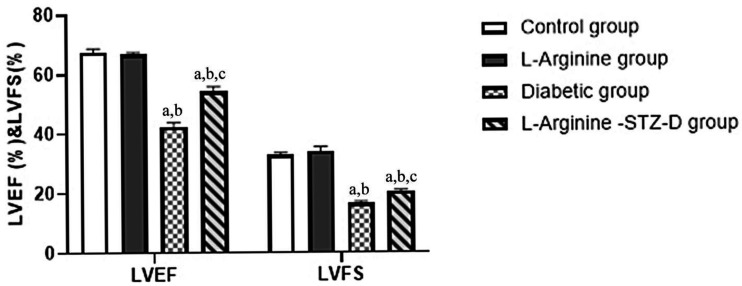
Echocardiographic parameters (LVFS % (left ventricular fractional shortening), LVEF % (left ventricular ejection fraction)) in the four groups. Mean ± SD is used to represent data. STZ-D; streptozotocin-induced diabetic. a: significant compared with controls, b: significant compared with L-Arginine group, c: significant compared with the Diabetic group. Significant means *p* value is less than 0.05.

**Table 1:** Body weight, cardiac and hemodynamic parameters in different studied groups.

	Control Group n = 10 rats	L-Arginine Group n = 10 rats	Diabetic Group n = 10 rats	L-Arginine-STZ-D Group n = 10 rats
FBW (gm)	296 ± 5.011	300.60 ± 11.118	310.30 ± 5.813 ^@#^	306.90 ± 10.181 ^@^
HW/BW (mg/gm)	0.0026 ± 0.0003	0.0028 ± 0.0003	0.0033 ± 0.0005 ^@#^	0.0030 ± 0.0002 ^@$^
Fasting Blood glucose(mg/dL)	107.40 ± 5.948	95.20 ± 9.818	583.10 ± 56.487 ^@#^	203.90 ± 24.429 ^@#$^
SBP (mmHg)	124 ± 6.041	91 ± 7.598 ^@^	123 ± 10.333 ^#^	84 ± 4.998 ^@#$^
DBP (mmHg)	72 ± 5.116	70 ± 7.187	82 ± 6.752 ^@#^	60 ± 3.062 ^@#$^
HR (beat/min.)	398.1 ± 3.665	288.9 ± 6.740 ^@^	466.3 ± 10.180 ^@#^	411.0 ± 9.684 ^@#$^
LVEF%	67.47 ± 1.245	66.67 ± 0.735	42.33 ± 1.350 ^@#^	54.12 ± 1.628 ^@#$^
LVFS%	32.81 ± 0.701	33.91 ± 1.587	16.55 ± 0.568 ^@#^	20.40 ± 0.541 ^@#$^

The data are shown as mean ± standard deviation. STZ-D (streptozotocin-induced diabetic), HR (heart rate), FBW (final body weight), LVEF % (left ventricular ejection fraction), LVFS % (left ventricular fractional shortening), SBP (systolic blood pressure), HW/BW (heart weight/body weight), DBP (diastolic blood pressure). ^@^: significant compared with controls; ^#^: significant compared with L-Arginine group; ^$^: significant compared with the Diabetic group. Significant means *p* < 0.05.

**Table 2:** Fold expression of microRNA-155b and m RNA of apoptotic genes, pro-inflammatory and anti-inflammatory cytokines in different studied groups.

	Control Group n = 10 rats	L-Arginine Group n = 10 rats	Diabetic Group n = 10 rats	L-Arginine-STZ-D Group n = 10 rats
microRNA-155b	1.059 ± 0.214	0.7185 ± 0.312	4.558 ± 1.524 ^@#^	2.462 ± 0.845 ^@#$^
BAX	0.3982 ± 0.126	0.3368 ± 0.105	1.187 ± 0.377 ^@#^	0.7119 ± 0.394 ^@#$^
BCL2	6.589 ± 0.254	7.139 ± 0.632 ^@^	5.020 ± 1.429 ^@#^	8.109 ± 1.481 ^@$^
IL1β	1.347 ± 0.165	0.7919 ± 0.145	3.049 ± 1.178 ^@#^	1.504 ± 0.283 ^#$^
IL6	0.5205 ± 0.213	0.3800 ± 0.473	4.954 ± 1.645 ^@#^	2.414 ± 0.796 ^@#$^
IL4	1.347 ± 0.165	1.379 ± 0.166	4.384 ± 0.615 ^@#^	2.705 ± 0.469 ^@#$^
IL13	1.504 ± 0.283	0.9282 ± 0.333	3.679 ± 1.340 ^@#^	2.745 ± 0.977 ^@#$^
TLR4	0.7802 ± 0.131	0.2284 ± 0.0891	4.239 ± 1.626 ^@#^	1.706 ± 0.227 ^@#$^

Mean ± SD is used to represent data. STZ-D; streptozotocin-induced diabetic. IL1β, 6, 4, 13 (interleukin 1β, 6, 4, 13) and TLR4 (toll like receptor-4). ^@^: significant compared with controls, ^#^: significant compared with L-Arginine group, ^$^: significant compared with the Diabetic group. Significant means *p* < 0.05.

**Table 3:** Correlations between TLR4, microRNA-155b expression, fasting blood glucose and myocardial expression of different studied genes.

		IL-1β	IL-6	IL-4	IL-13	TLR4	BAX	BCL2
Blood glucose	r p	0.777 ***	0.853 ***	0.902 ***	0.740 ***	0.867 ***	0.781 ***	−0.581 ***
microRNA-155b	r p	0.703 ***	0.779 ***	0.801 ***	0.763 ***	0.842 ***	0.697 ***	−0.293 ***
TLR4	r p	0.633 ***	0.864 ***	0.827 ***	0.731 ***			

IL-1β, 6, 4, 13 (interleukin 1, 6, 4, 13) and TLR4 (toll-like receptor-4) *** *p* < 0.001.

**Table 4:** Area percentages of PAS and collagen deposition among studied groups.

	Groups	Control Group	L-Arginine Group	Diabetic Group	L-Arginine-STZ-D Group	*p*-Value
Parameters						
Area % collagen (mean ± SD)		6.62 ± 0.8	6.03 ± 0.4 ^@^	20.00 ± 1.5 ^@#^	12.83 ± 0.8 ^@#$^	<0.0001 *
Area % PAS (mean ± SD)		24.37± 1.0	16.01± 1.6 ^@^	29.97± 1.7 ^@#^	20.29 ± 1.1 ^@#$^	<0.0001 *

SD: standard deviation. STZ-D: streptozotocin-induced diabetic. *: significant difference *p* < 0.05. ^@^ significant compared to controls. ^#^ significant compared to the l-arginine group. ^$^ significant relative to the diabetic rats.

**Table 5:** Primers of mRNA of apoptotic genes, pro-inflammatory and anti-inflammatory cytokines, miR155 and their reference genes.

Primer Name	Primer Sequence
microRNA155b	F: 5′-GACTGTTAATGCTAATCGTGATAG-3′ R: 5′-GTGCAGGGTCCGAGGTATTC-3′
U-6	F: 5′-CGCTTCGGCAGCACATATAC-3′ R: 5′-TTCACGAATTTGCGTGTCAT-3′
TLR4	F: 5′-TGACGCCTTTGTTATCTACTCC-3′ R: 5′-GGTCTGGGCAATCTCATACTC-3′
IL6	F: 5′-TCTACGTCCATCAGGAGCCA-3′ R: 5′-TTGGGCAATAGGCAGCATCA-3′
IL1β	F: 5′-GCTCTAGTTCAGGGCAGGCA-3′ R: 5′-TTTCTGTTTGGGGTTCCACTCA-3′
1L4	F: 5′-GTGAGTTCAGACCGCTGACA-3′ R: 5′-TCACGCTCACACTCAGACAC-3′
IL13	F: 5′-AGTCCTGGAGCTGAGAGAGG-3′ R: 5′-GTAGACCCCCATCCTCCTGT-3′
BAX	F: 5′-ATGGACGGGTCCGGG GAG-3′ R: 5′-ATCCAGCCCAACAGCCGC-3′
BCL2	F: 5′-AAGCCGGCGACGACTTCT-3′ R: 5′-GGTGCCGGTTCAGGTACTCA-3′
GAPDH	F: 5′-GGATTTGGTCGTATTGGG-3′ R: 5′-GGAAGATGGTGATGGGATT-3′

IL1β, 6, 4, 13 (interleukin 1β, 6, 4, 13), TLR4 (toll-like receptor-4), and apoptotic markers (BAX, BCL2). F: forward; R: reverse.

## Data Availability

The original contributions presented in this study are included in the article. Further inquiries can be directed to the corresponding author.
